# Assessment of nicotine dependence among smokers in Nepal: a community based cross-sectional study

**DOI:** 10.1186/s12971-015-0053-8

**Published:** 2015-08-26

**Authors:** Umesh Raj Aryal, Dharma Nand Bhatta, Nirmala Shrestha, Anju Gautam

**Affiliations:** Department of Community Medicine, Kathmandu Medical College, Sinamangal, Kathmandu Nepal; Faculty of Medicine, Epidemiology Unit, Prince of Songkla University, Songkhla, Thailand; Department of Public Health, Nobel College, Pokhara University, Sinamangal, Kathmandu Nepal

**Keywords:** Current smoking, FTND, HSI, Nicotine dependence

## Abstract

**Background:**

The Fagerström Test for Nicotine Dependence (FTND) and Heaviness of Smoking Index (HSI) are extensively used methods to measure the severity of nicotine dependence among smokers. The primary objective of the study was to assess the nicotine dependence amongst currently smoking Nepalese population.

**Methods:**

A community based cross-sectional study was conducted between August and November 2014. Information was collected using semi-structured questionnaire from three districts of Nepal. Data on demographic characteristics, history of tobacco use and level of nicotine dependence were collected from 587 smokers through face to face interviews and self-administered questionnaires. Non-parametric test were used to compare significant differences among different variables.

**Results:**

The median age of respondents was 28 (Inter-Quartile Range: 22–40) years and the median duration of smoking was 10 (5–15) years. Similarly, the median age for smoking initiation was 16 (13–20) years and the median smoking pack year was 4.2 (1.5–12). One third of the respondents consumed smokeless tobacco products. Half of the respondents wanted to quit smoking. The median score for FTND and HSI was 4 (2–5) and 2 (0–3) respectively. There was significant difference in median FTND score with place of residence (*p* = 0.03), year of smoking (*p* = 0.03), age at smoking initiation (*p* = 0.02), smoking pack year (*p* < 0.001) and consumption of smokeless products (*p* < 0.05). Similarly, there was also significant difference in median HSI score with year of smoking (*p* = 0.002), age of smoking initiation (*p* < 0.001), smoking pack year (*p* < 0.001), and consumption of smokeless products (*p* < 0.05). As per FTND test score, two in ten current smokers had high nicotine dependence (FTND > 6), and HSI scored that three in ten current smokers had high nicotine dependence (HSI > 3).

**Conclusion:**

Our finding revealed that nicotine dependence is prevalent among Nepalese smoking population. Further studies are required for assurance of tools through bio-markers. Next, smoking cessation program need to be developed considering level of nicotine dependence and pattern of tobacco use.

## Background

Nicotine is highly addictive chemical found in tobacco that makes one difficult to stop smoking once initiated, and this property of nicotine is similar to those of heroin and cocaine [1]. Hence, nicotine dependence is a substance related disorder which is an obstacle in the smoking cessation among smokers [2]. Nicotine addiction is the cause of premature death for one person in every six seconds and 80 % of them are from the low- and middle income countries (LMICs) [3]. It kills more than 15,000 people annually in Nepal of which 40 % are female [4].

Recent study finds that nearly 16 % of Nepalese population (15–69 years) are currently smoking of them 85 % are daily smokers [5]. Among current daily smoking population, 28.7 % were found to be smoking < 5 cigarettes, 40 % smoked 5–9, 16.9 % smoked 10–14, 13.5 % smoked 15–24 and the remaining percentage smoked ≥ 25 cigarettes per day. Three out of ten current smokers tried to quit cigarettes and two out of ten were advised by doctors to stop smoking [5]. However, there is paucity of data that explored the proportion of nicotine dependence in general population in Nepal and as a result there has not been any smoking cessation interventions effectively implemented in community and in hospitals yet.

Fagerström Test Nicotine Dependence (FTND) was introduced in 1991 that corrected inaccuracies of earlier measures and reduced number of questions from 8 to 6 without losing reliability [6–10]. The FTND is considered to be a self-reporting tool which conceptualizes dependence through physiological and behavioral symptoms [11]. Similarly, heaviness of smoking index (HSI) which is a subset of FTND and it can be an appropriate alternative tool to FTND to measure nicotine dependence [12]. HSI is comprised of two items: time to smoking first cigarette after waking up and the number of cigarettes smoke per day [12]. Several populations based studies have explained that HSI has high consistency compared to FTND [11, 13, 14].

Despite the fact, majorities of the public health studies focused only on psychological and behavioral factors associated with tobacco smoking. The assessment of nicotine dependence is one of the important areas of public health which has not been realized yet in the LMICs like Nepal. Therefore, the present study was aimed to assess the nicotine dependence among Nepalese current smoking population.

## Methods

### Study design and site

A community based cross-sectional study was conducted from August to November 2014. This study was carried out in three districts of Nepal viz. Kathmandu, Lalitpur and Morang. The study was conducted in urban settings in Kathmandu and in Morang districts; however, it was conducted in rural settings in Lalitpur district. Kathmandu is the capital of Nepal and Lalitpur district is adjacent to Kathmandu situated in the central part of the country whereas Morang district is situated in the eastern terai region of Nepal which is 397 km south-east from other two study districts. Kathmandu metropolis of Kathmandu district, Biratnagar sub-metropolis of Morang district and Bungmati and Khokana village development committees of Lalitpur district were purposively selected for the study, and in all the settings, all the people have easy access to all type of tobacco products.

### Study population and sampling method

The study population comprised of current smokers with age above 15 years and willing to participate in the study. There was an unavailability of sampling frame for current smoking population in the study site as well as elsewhere in Nepal. Therefore, a purposive sampling technique was used to collect the information. In the rural area, undergraduate medical students purposively visited the households during their family health project and selected the respondents. The respondents were also selected from the teashops and other public places where people gathered (“*Chowk*” in Nepalese language). In the urban area, public health graduates were purposively selected the respondents from the coffee shop, cyber café (internet surfing zone) and parks.

### Sample size

A total of 600 respondents were included in the study: Kathmandu (*n* = 301), Lalitpur (202) and Morang (97) districts. Further, the study had planned to incorporate at least 200 samples from each area but could only take 97 respondents from Biratnagar because of resource constraints. Therefore, 301 respondents (101 extra respondents) were incorporated from Kathmandu metropolis. Thirteen respondents from the urban settings were excluded from the analysis due to their response “I do not know” in the FTND questionnaire. Final analysis was done among 587 respondents and the overall response rate was 98.7 %.

### Tools and study variables

The information was collected through both face to face interviews and self-reported (20 % respondents in urban site) semi-structured questionnaire. Both English (by medical students) and Nepalese (by public health students) versions of questionnaires were used for data collection. Nepalese version of the questionnaire was further translated in English ensuring that the meaning of questions remains unchanged. The semi-structured questionnaire included three sections as followings:

- Demographic profile: it contained information on age, sex, caste, residents and district. Further, the caste was classified using Nepal government classification [15].
- Smoking behavior: It contained information about *the* number of cigarette smoked per day, age at smoking initiation, duration of the smoking, smokeless tobacco products used (yes/no), ever tried to quit smoking (yes/no) and the number of times tried to quit in last one year. Next, we computed the number of pack-years using the following formula to correlate with nicotine dependence: ([number of cigarettes smoked per day X number of years smoked] /20, 1 pack = 20 cigarettes).
- Nicotine Dependence: It contained following six standard questions of the Fagerström Tolerance of Nicotine Dependence (FTND) [6]. i. How soon after you wake up do you smoke your first cigarette? [within 5 min (3 points), within 6–30 min (2 points), within 31–60 min (1 point); after 60 min (0 point)]; ii. Do you find it difficult to refrain from smoking in places where it is forbidden? [yes (1 point), no (0 point)]; iii. Which cigarette would you hate most to give up? [The first one in the morning (1 point), any other (0 point)]; iv. How many cigarettes per day do you smoke? [10 or less (0 point), 11–20 (1 point), 21–30 (2 points), and 31 or more (3 points)]; v. Do you smoke more during the first hours after waking than during the rest of the day? [Yes (1 point), no (0 point)]; vi. Do you smoke even when you are ill enough to be in bed most of the day? [Yes (1point), no (0 point)]. A total score for Nicotine dependence (FTND) were obtained by summing above given points where minimum = 0 and maximum score = 10. Maximum total score for HSI was 6 which were computed by summing item i and iv [12] .

### Statistical analyses

All the collected data were entered into Microsoft Excel (2007 version) and SPSS (20.0 version) was used for analysis. Data were cleaned and checked for disparities both before and after the data entry. Descriptive statistics (percentage, median, inter-quartile range [IQR]) was used to demonstrate demographic and nicotine dependence characteristics of respondents. Both FTND and HSI score were classified as very low/none, low, medium and high nicotine dependence using proposed cut-off points [10–16].

Chi-square test was used to compare proportion difference between categorical variables. Spearman rank correlation was used to measure the relationship between nicotine dependence (FTND and HSI Score) and smoking behaviors (years of smoking, number of pack-years and age at smoking initiation). We applied Mann-Whitney U test and Kruskal-Wallis test to compare the median difference in FTND as well as HSI scores with demographic characteristics and smoking behavior related variables because most of the numerical data were skewed in nature.

### Ethical consideration

Institutional Review Committee of Kathmandu Medical College approved this study. Prior to interview, the objectives of the study were explained to the respondents, informed them that their participation was voluntary, responses would be kept confidential, and respondents’ privacy would be maintained. Written (for literate) and verbal (for illiterate) consents were obtained from the respondents before they were interviewed.

## Results

The study finds 587 currently smoking individuals out of which 15.2 % were female. Four out of ten respondents were from upper caste group, two out of ten were relatively disadvantaged group, three out of ten were relatively advantaged group and one in ten was from the remaining groups (disadvantages non-dalit terai caste groups, indigenous and socially disadvantaged groups, and religious minorities groups). More than one third (37 %) of the respondents were between 15 and 24 years and less than 5 % were aged 65 years and more. The median age of current smoking is 28 (IQR: 22–40) years. There were significant differences between sex and age of the smokers (*p* = 0.001) (Table 1).

**Table 1:** Socio-demographic characteristics of current smoking respondents by sex (*n* = 587)

Variable	Male (*n* = 498)	Female (*n* = 89)	Total (*n* = 587)	*p* value
Age (years)				
15–24	195 (39.2)	22 (24.7)	217 (37)	χ2 = 19.7 0.001
25–34	146 (29.3)	19 (21.3)	165 (28.1)	
35–44	70 (14.1)	18 (20.2)	88 (15)	
45–54	35 (7.0)	15 (16.9)	50 (8.5)	
55–64	30 (6.0)	10 (11.2)	40 (6.8)	
65 and above	22 (4.4)	5 (5.6)	27 (4.6)	
*Caste* ^*a*^				
Upper caste groups	198 (39.8)	26 (29.2)	224 (38.2)	χ2 = 6.6 0.19
Relatively advantaged groups	154 (30.9)	36 (40.4)	190 (32.4)	
Relatively disadvantaged groups	106 (21.3)	17 (19.1)	123 (21.4)	
Disadvantages non-dalit terai caste groups	17 (3.4)	3 (3.4)	20 (5.1)	
Indigenous and socially disadvantaged groups	23 (4.6)	7 (7.9)	30 (3.4)	
*Resident* ^*b*^				
Urban	339 (68.1)	59 (66.3)	398 (67.8)	χ2 = 0.11 0.74
Rural	159 (31.9)	30 (33.7)	189 (32.2)	

^a^Upper caste (Brahmin, Chhetri); Relatively advantaged group (Newar and Gurung); Indigenous disadvantaged groups (Magar, Tamang, and Rai/Limbu); Disadvantages non-dalit terai caste (Yadav and Thakur ); Indigenous and socially disadvantaged (dalit) [15]

^b^Urban area (Kathmandu metropolitans, Biratnagar municipalities); Rural area (Bungamati and Khokana villages of Lalitpur district)

Overall median age at smoking initiation was 16 (IQR: 15–20) years. The median age at smoking initiation for both male and female was 16 respectively with (IQR: 15–20) and 16 (IQR: 14–20) years. Nearly 93 % of the respondents smoked first cigarette before their 25th birthday. Among them, 60 % began smoking before their 18th birthday and nearly 8.6 % began smoking before 11 years of age. The median number of cigarettes smoked per day was nine (IQR: 5–15). The median duration of cigarette smoking was 10 (IQR: 5–15) years. Median number of cigarette pack year was 4.2 (IQR: 1.5–12). More than half (51.4 %) of the respondents have ever tried to quit smoking and 49.4 % tried to quit smoking last year. The median number of times they tried to quit was two (IQR: 1–4). Along with smoking, one third (34.2 %) of the respondents consumed smokeless tobacco product of which 74.1 % were from urban area and 12.1 % were female (data not shown in table).

Two out of ten respondents smoked within five minutes after waking, four out of ten smoked after 60 min of waking, nearly half of the smokers notified that they had difficult to refrain from forbidden places, five out of ten would hate to give up first cigarette in the morning, four out of ten respondents have smoked more than ten cigarettes per day, less than 5 % of the respondents have smoked more than 30 cigarettes per day, more than half of the respondents have smoked more frequently during the first hour of waking and four out of ten respondents reported that they have smoked during sickness. The percentage of responses for FTND/HSI tests did not differ significantly between male and female (*P* > 0.05) (Table 2). In overall, median FTND and HSI score was 4(IQR: 2–5) and 2 (0–3).

**Table 2:** Percentage (95 % CI) of males and females who responded to FTND categories

Items	Male (*n* = 498)	Female (89)	Total (587)	*p* value
Item 1				
Time for first cigarette after wake up :>60 min (0) ^a^	39.6 (35.3;43.9)	33.7 (28.8;38.6)	38.7 (33.8;43.6)	χ2 = 5.40 P = 0.15
Time for first cigarette after wake up :31–60 min (1) ^a^	18.1 (14.6;21.6)	14.6 (10.9;18.3)	17.5 (13.4;21.6)	
Time for first cigarette after wake up :6–60 min (2) ^a^	18.7 (15.4;22.0)	29.2 (24.6;33.8)	20.3 (16.2;24.4)	
Time for first cigarette after wake up : ≤ 5 min (3) ^a^	23.7 (19.9;27.4)	22.5 (18;27)	23.5 (18.9;28.0)	
Item 2				
Difficult to refrain from smoking in places where it is forbidden (1) ^b^	48.2 (43.7;52.7)	40.4 (35;45.8)	47 (41.9;52.1)	χ2 = 1.8 *P* = 0.18
Item 3				
Cigarette would you hate most to give up first in the morning (1) ^b^	50.6 (46.1;55.1)	55.1 (49.9;60.3)	51.3 (46.2;56.4)	χ2 = 0.59 *P* = 0.44
Item 4				
Cigarette per day: 1–10 (0) ^a^	61.6 (57.3;65.9)	59.6 (54.6;64.6)	61.3 (56.4;66.2)	χ2 = 0.55 *P* = 0.98
Cigarette per day: 11–20 (1) ^a^	28.1 (24.2;32.2)	31.5 (26.6;36.4)	28.6 (24.1;33.1)	
Cigarette per day: 21–30 (2) ^a^	5.8 (3.6;7.9)	5.6 (3.2;8)	5.8 (3.5;8.2)	
Cigarette per day: 31–30 (3) ^a^	4.4 (2.6;6.2)	3.4 (1.5;5.3)	4.3 (2.7;5.9)	
Item 5				
Smoke more frequently during the first hours of waking (1) ^b^	54 (49.7;58.3)	44.9 (39.6;50.2)	52.6 (47.9;57.3)	χ2 = 2.59 *P* = 0.11
Item 6				
Smoke if you are so ill that you are in bed (1) ^b^	41.4 (37.1;45.7)	40.4 (35;45.8)	41.2 (36.7;45.7)	χ2 = 0.19 *P* = 0.91

Parenthesis is the given score for each answer. (1) Indicates “yes” responses in item 2, 5, and 6 and first cigarette of day in item 3

^a^Both FTND and HSI

^b^ = FTND

There were no significant differences in median FTND and HSI scores with age, sex and caste (*P* > 0.05). Next, there was significant difference in median FTND score with place of residence (*p* = 0.03), the year of smoking (*p* = 0.03), age of smoking initiation (*p* = 0.02), smoking pack year (*p* < 0.001) and consumption of smokeless products (*p* < 0.05). Similarly, there was significant difference in Median HSI score with year of smoking (*p* = 0.002), age of smoking initiation (*p* < 0.001), smoking pack year (*p* < 0.001), and consumption of smokeless products (*p* < 0.05) (Table 3).

**Table 3:** Distribution of FTND and HSI total score for smokers based on the background characteristics and smoking behaviors

Variable	FTND	*p*-value	HSI	*p*-value
	Median (IQR)		Median (IQR)	
Sex *				
Male	4 (2–5)	0.92	2 (0–3)	0.38
Female	3 (2–5)		2 (1–3)	
Age (years) **				
15–24	4 (2–5)	0.91	1 (0–3)	0.48
25–34	3 (2–5)		2 (0–3)	
35–44	3 (2–5)		2 (1–3)	
45–54	4 (3–6)		2 (1–3)	
55–64	4 (3–5)		2 (1–3)	
65 and above	4 (2–6)		2 (1–3)	
Caste **				
Upper caste groups	4 (2–5)	0.51	1 (0–3)	0.27
Relatively advantaged groups	3 (2–5)		2 (0–3)	
Relatively disadvantaged groups	4 (2–5)		2 (1–3)	
Disadvantages non-dalit terai caste groups	4 (2–6)		1 (0–3)	
Indigenous and socially disadvantaged groups	4 (3–6)		1 (0–4)	
Resident *				
Urban	3 (2–5)	0.03	2 (1–3)	0.37
Rural	4 (2–5)		2 (0–3)	
Year of smoking (median year) *				
<10	3 (2–5)	0.03	1 (0–3)	0.002
≥10	4 (2–5)		2 (1–3)	
Age of smoking initiation (median age) ^*^				
<16	4 (3–5)	0.02	2 (1–3)	*p* < 0.001
≥16	3 (2–5)		1 (0–3)	
Smoking pack year ( median pack year) *				
<4.2	3 (2–5)	*p* < 0.001	1 (0–2)	*p* < 0.001
≥4.2	4 (3–5)		1 (1–3)	
Consumption of smokeless products				
Yes	4 (3–6)	*p* = 0.002	2 (1–3)	*p* = 0.004
No	3 (2–5)		1 (0–3)	

P values were obtained either by Mann–Whitney Test (*) or Kruskal Wallis Test applied (**)

Based on FTND score, three out of ten respondents had very low nicotine dependence, nearly two out of ten had low, three out of ten had medium, and two out of ten had high dependence (Table 4). Next, based on HSI score, two out of ten had none dependence, two out of ten had low, nearly two out of ten had medium and three out of ten had high dependence. There was no significant difference in proportion of level of nicotine dependence among male and female respondents for both FTND (*p* = 0.95) and HSI (*p* = 0.13) (Table 4). Further, the respondents who were smokeless tobacco user had higher nicotine dependence compared to user of smoking tobacco in both FTND and HSI (FTND ≥4: 42.3 % vs.32.1 %, χ2 = 10.1, *p* = 0.04; HSI ≥ 3: 43.3 % vs.30.0 %, χ2 = 12.3, *p* = 0.02).

**Table 4:** Classification of FTND and HSI score based on quartile values by sex

FTND classification	Male (*n* = 498)	Female (*n* = 89)	Total (*n* = 587)	*p* value
	% (95 % CI)	% (95 % CI)	% (95 % CI)	
Very low (0–2)	31.9 (27.8;36.2)	31.4 (22.0; 42.2)	31.9 (28.4;36.1)	
Low (3–4)	16.2 (13.1;19.8)	21.3 (13.3;31.3)	17.1 (14.1;20.3)	
Medium (5)	31.9 (27.3;35.6)	24.7 (16.1;31.9)	30.3 (26.6;34.2)	χ2 = 2.4 *p* = 0.5
High (6–10)	20.4 (17.1;24.3)	22.4 (14.2;32.5)	20.4 (17.2;23.8)	
HSI classification				
None (0)	27.1 (22.3;29.6)	19.1 (11.5;28.8)	25.9 (22.4;29.6)	
Low (1)	21.1 (18.5;25.4)	25.8 (17.1;36.2)	21.8 (18.5;25.4)	χ2 = 4.1 *p* = 0.23
Medium (2–3)	16.9 (14.7;21.1)	22.5 (14.3;32.5)	17.7 (14.7;21.1)	
High (4–6)	34.9 (30.7;39.3)	32.5 (23.1;43.3)	34.6 (30.7;38.5)	

Median (IQR) score for FTND and HSI was 4 (2–5) and 2 (0–3) respectively

## Discussion

Nepal is a country having multi-ethnic and multi-cultural diversities where smoking prevalence is high and vary with demographic characteristics [17]. Despite high smoking prevalence, none of the scientific studies have explored about level of nicotine dependence among smokers in Nepal. It is essential for stakeholders to understand the nature of nicotine addiction among smokers for successful implementation of smoking cessation programs. Lack of better understanding on level of nicotine dependence might be the obstacle for choosing appropriate cessation strategies [18].

Our study would, in some extent, fill the gap in the literature related to nicotine dependence among smokers in Nepal. Our analysis explored nicotine dependence of current smoker people living in both urban and rural areas of Nepal. Both FTND and HSI methods were applied to measure the level of nicotine dependence which was found inconsistent with previous studies [14, 18, 19]. One of the possible reasons for inconsistency might be because of the different cut-off points in both FTND and HSI. In addition, it might also depend on the pattern of tobacco use as well. To confirm nicotine dependence among smoker, future studies needs to correlate between biomarkers [20, 21] of exposure to cigarette smoke and responses to specific items of the FTND and HSI.

Our study revealed that those respondents who smoked and used smokeless tobacco products have high nicotine dependence than those who smoked only. These findings were similar with Indian studies that revealed higher percentage of nicotine dependence was associated with users of tobacco in mixed form [22, 23]. Many people in south Asia believed that smokeless tobacco products are less harmful and cheaper than cigarettes. Lack of knowledge and wrong perception about benefits of chewing products might be one of the reasons for using smokeless tobacco products [24]. Nearly one third of the respondents reported that they felt difficulties to smoke in public places and consumed smokeless tobacco products. This could also be the reason to consume smokeless tobacco. Further, research on addiction of smokeless tobacco products is necessary for low- and middle-income countries including Nepal.

Our data revealed that the level of nicotine dependence were associated with smoking pattern such as year of smoking, age at smoking initiation and smoking pack years. This finding was found similar with other population based studies from India and the Netherlands [18, 25]. This study revealed no association between demographic characteristics and nicotine dependence which was found inconsistent with previous studies [18, 26]. The difference might be due to small sample size in sub-categories. Previous studies from India and Singapore explained that the level of nicotine dependence were associated with age, sex and ethnicity [18, 26]. Therefore, understanding the level of nicotine dependence through stratification/cluster sampling of demographic characteristics including occupation, education and socio-economic status are essential in the places where the smoking is prevalent [18].

Nepal demography and health survey 2011 revealed that the large frequency of tobacco consumption were found in Nepalese rural community [17], and similar findings were shown by this study that the rural community had higher score of nicotine dependence in comparison to urban community. The study from India also showed the link between tobacco consumption and psychological dependence in rural population [27]. However, it could be imprudent to conclude that the relationship between nicotine dependence and smoking frequency among rural community because of the smaller sample size from rural community in our study.

Our study revealed that the age at smoking initiation was during adolescence. Many adolescents failed to understand addictive nature of smoking and increased risk of becoming smokers [25, 28, 29]. In the later stage of their age, they are hooked to nicotine and finds it difficult to quit smoking [30]. Our results provided further empirical support that smokers had tried to quit smoking several times but they failed. This evidence supports the urgent need to develop smoking cessation intervention programs in Nepalese community.

The baseline information related to smoking and nicotine dependence are crucial for policy makers and implementer to implement effective tobacco control programs [18]. Encouraging tobacco users to quit or cessation is best approach to reduce tobacco-related disease, deaths and health care costs [31]. The following 5 A’s approach: Ask (identify smoking/tobacco use status); Advise (advise patient to quit); Assess (determine interest/readiness to quit); Assist (in developing quit plan if ready to plan, otherwise motivate them to quit); Arrange (follow-up contact)] would be appropriate for motivation to quit smoking [31]. Based on our study, behavioral changed intervention at community clinics managed by trained medical and public health professional can be an effective program for smoking cessation.

The study has covered the same strength as other studies. First, our study sample was representative of different geographical locations of Nepal. Second, sample size was sufficient to measure relationship between variables. Third, the data provided the important information for feasibility of FTND in Nepalese context. Despite above strengths, we have identified some limitations in this study. First, the cultural and geographical variation might affect level of nicotine dependence [32, 33]. Second, the study was based on non-probability sampling. Third, there was a possibility of information bias specially related to history of smoking and number of cigarettes smoked per day. Fourth, HSI was subset of FTND items, thus comparison between items might leads to false conclusion. Thus our study recommends comparison between FTND and HSI will be suitable with other instruments.

## Conclusions

Our finding revealed that nicotine dependence is prevalent among Nepalese smoking population. Further studies are required to conduct for assurance of tools through bio-markers. Next, smoking cessation programs need to be developed and implement considering the level of nicotine dependence and pattern of tobacco use.

## Abbreviations

FTND: The fagerström test for nicotine dependence
HSI: Heaviness of smoking index
IQR: Inter-quartile range
